# Nutraceutical Potential of Bitter Melon (*Momordica charantia*) on Cancer Treatment: An Overview of In Vitro and Animal Studies

**DOI:** 10.3390/cimb47060425

**Published:** 2025-06-06

**Authors:** Georgia-Eirini Deligiannidou, Agathi Pritsa, Anastasios Nikolaou, Efthymios Poulios, Christos Kontogiorgis, Sousana K. Papadopoulou, Constantinos Giaginis

**Affiliations:** 1 Department of Nutritional Sciences and Dietetics, School of Health Sciences, International Hellenic University, 57001 Thessaloniki, Greece; edeligia@med.duth.gr (G.-E.D.); souzpapa@gmail.com (S.K.P.); 2 Department of Medicine, School of Health Sciences, Democritus University of Thrace, 68100 Alexandroupolis, Greece; ckontogi@med.duth.gr; 3 Department of Molecular Biology and Genetics, School of Health Sciences, Democritus University of Thrace, 68100 Alexandroupolis, Greece; anikol@mbg.duth.gr; 4 Department of Food Science and Nutrition, School of Environment, University of Aegean, 81100 Myrina, Greececgiaginis@aegean.gr (C.G.)

**Keywords:** natural products, bitter melon, *Momordica charantia*, in vitro, cancer, anti-inflammatory, animal model, nutraceuticals

## Abstract

Bitter melon (*Momordica charantia*) has been extensively investigated for its potential in cancer treatment. In this work, we provide an overview of in vitro and animal studies exploring its bioactive compounds, extracts, extracellular vesicles, fusion proteins, co-treatment with conventional pharmaceuticals, and utilization of nanoparticles, demonstrating promising cytotoxic and apoptotic effects across various cancer cell lines. A comprehensive search of online databases, e.g., PubMed, Scopus, and Web of Science, and Google Scholar was performed in the last decade, utilizing relevant keywords and applying several inclusion and exclusion criteria. The plant and its derivatives exhibit significant antiproliferative properties and modulate key signaling pathways. Additionally, animal studies have validated its antitumor potential, highlighting its ability to suppress tumor growth, modulate immune responses, and enhance chemotherapeutic efficacy in vivo. Although several compounds of the plant have been investigated, the insights regarding their mechanisms of action remain limited. Also, plant-derived extracellular vesicles show promise as natural carriers for targeted drug delivery, while fusion proteins improve cellular uptake and apoptosis induction. Finally, the integration of bitter melon components into nanomedicine underscores their potential for advanced therapeutic applications. Collectively, these findings reinforce the growing interest in utilizing bitter melon-derived compounds for cancer treatment and signal the need for further research to optimize their clinical translation.

## 1. Introduction

*Momordica charantia* (MC), commonly known as bitter melon, is a traditional medicinal plant found in various countries, which has been investigated in the treatment of diseases such as diabetes, inflammation, and cancer [1,2,3]. Previous research indicates that bitter melon extracts or isolated compounds, such as triterpenoids and saponin compounds, have an impact on glycemic control and metabolic syndrome. As demonstrated by the outcomes of in vitro and animal studies reporting reductions in mRNA and protein expression levels of key genes involved in fatty acid biosynthesis and glycolysis rate, there is evidence of MC’s potential on the modulation of glucose homeostasis and lipid metabolism [4,5,6,7,8]. Notably, several studies have also aimed to investigate the potential cardioprotective properties in the setting of cancer treatments where cardiotoxicity is produced [9]. Bitter melon extracts and isolated bioactive compounds have also been studied for their antibacterial, antioxidant, and anti-inflammatory effects in the setting of in vitro and animal studies. Namely, plumericin, an iridoid lactone of the plant, has demonstrated antibacterial activity against *Enterococcus faecalis* and *Bacillus subtilis* as well as inhibitory potential in the proliferation of acute and chronic leukemic cancer cells [10]. The antioxidant potential of plant extracts, related to the induction of reactive oxygen species (ROS) and cellular damage, has been an item of investigation as a crucial attribute that is often linked to the anti-inflammatory properties of the extracts, particularly in the setting of in vitro studies. Previous investigations on the antioxidant potential of ethanol, methanol, and aqueous extracts of MC in vitro have reported improved cell viability and antiproliferative effects, high free radical scavenging potential, a reduction in the H₂O₂-induced apoptosis, and lipopolysaccharide (LPS)-stimulated NO production in various cell lines [4,11,12,13]. Additionally, evaluations related to stress response and inflammation have linked the MC extract and isolated compounds’ activities to the suppression of the MAPK (mitogen-activated protein kinase signaling) pathway and the inhibition of inducible NO synthase (iNOS), cyclooxygenase (COX)-2, tumor necrosis factor-α (TNF-α), interleukin-1β (IL-1β), IL-6, IL-8, IL-23a, macrophage inflammatory protein-1 alpha (MIP-1α), and monocyte chemoattractant protein (MCP-1), as well as nuclear factor kappa B (NF-κB) expression levels [13,14,15].

Besides the plant’s key bioactive compounds like cucurbitane-type triterpenoids and alpha-momorcharin (α-MMC), which have reportedly shown significant cytotoxic, antitumor, and immunomodulatory properties, other compounds, such as α-spinasterol, stigmasterol, campesterol, and stigmasta-7,25-dien-3beta-ol, have also displayed non-toxic and drug-like properties [16], while the triterpenoid, 5β,19-epoxy-19-methoxycucurbita-6,23-dien-3β,25-diol has also been identified as a peroxisome proliferator-activated receptor-γ (PPAR-γ) activator in breast cancer MCF-7 cells [17]. In a similar setting, the BG-4 bioactive peptide isolated from MC has also demonstrated anti-inflammatory properties via the inhibition of IL-6 and TNF-α production in LPS-activated macrophages [18], also associated with reduced nuclear translocation of the p65 NF-κB subunit [19] and linked to reduced expression of anti-apoptotic beta cell lymphoma-2 (Bcl-2) proteins and increased expression of Bax [20].

The plant is being studied further for its nutraceutical and pharmacological benefits, especially for its role in metabolic reprogramming and managing chronic human diseases [21]. Recent studies utilizing network pharmacology have shed light on the therapeutic potential of bitter melon’s key bioactive compounds, including momordicoside K, kaempferol, and quercetin. These compounds are closely associated with anti-BRCA mechanisms, operating through pathways such as PI3K/AKT signaling, transcriptional regulation, and calcium signaling. Such findings offer valuable insights into the molecular underpinnings of bitter melon’s anticancer properties and underscore its promising applications in breast cancer therapy [22].

In an effort to summarize its established pharmacological potential and highlight key challenges, this review explores the role of bitter melon (*Momordica charantia*) in cancer treatment, in the setting of in vitro and animal studies.

## 2. Materials and Methods

A literature review was conducted, including studies published from 2015 to 2024 on accurate scientific databases, e.g., PubMed, Scopus, and Web of Science, using the following search terms: (“Momordica charantia” OR “bitter melon”) AND (“cancer” OR “tumor”). Publicly available research articles were screened based on the criteria described in Table 1. All eligible papers were screened by two reviewers in terms of “Title” and “Abstract” relevance and then classified per the study design (in vitro studies and animal studies). At this stage, articles were excluded when the study design was one other than that predefined in the inclusion criteria. Then, the full text of the included papers was screened, and the papers that met the inclusion criteria were distributed to the authors for data extraction. At this stage, articles reporting outcomes other than the ones predefined in the inclusion criteria were excluded. The retrieved studies were also checked for relevant studies cited in their manuscript.

Based on the initial screening of full texts, the authors agreed to stratify the included articles per the study design instead of cancer type evaluated, given that the majority of studies targeted the cell viability and toxicity of the interventions and the inhibitory potential of key signaling pathways of inflammation, autophagy, and cytokine expression, in an effort to provide an holistic view of the current knowledge at each evaluation stage—i.e., in vitro and animal models.

## 3. Results

### 3.1. In Vitro Studies

**Figure 1:**
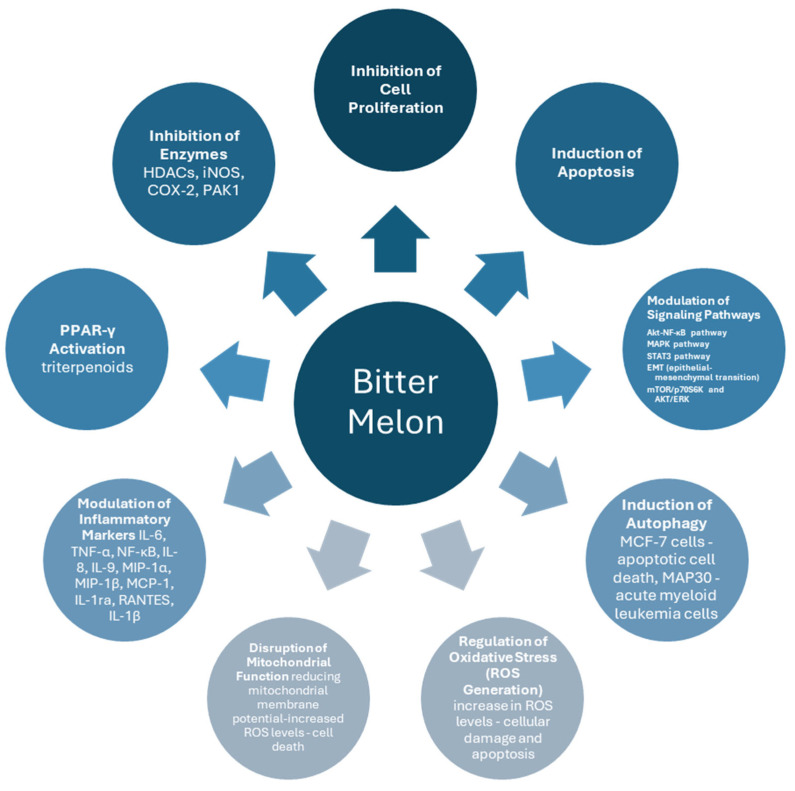
Key mechanisms identified in in vitro studies.

The investigation of the current literature on in vitro studies, particularly targeting research on cancer cells, resulted in 35 studies on bitter melon and its derivatives. Overall, the findings highlighted the diverse bioactive properties of this natural product across various cancer cells. Key compounds found in bitter melon, such as 3β,7β,25-trihydroxycucurbita-5,23(E)-dien-19-al and cucurbitane-type triterpenoids, have reportedly exhibited antiproliferative effects by several modulating signaling pathways, including Akt-NF-κB, p38 MAPK, and STAT3, while triterpenoids—a family closely researched—including compounds such as karaviloside III, kaguaovin L, and kaguacidine A, have reportedly exhibited cytotoxicity and anti-inflammatory activity in various cancer cell lines. Additionally, proteins such as alpha-momorcharin (α-MMC) and Momordica anti-HIV protein (MAP30) were among the bitter melon derivatives closely studied for their ability to induce apoptosis, promote ROS generation, regulate autophagy, and impact cytokine expression. Also, research on bitter melon extracts (including organic solvents and aqueous extracts) and juices has demonstrated the potential to enhance chemotherapy sensitivity, suppress proliferation, disrupt mitochondrial function, and modulate key pathways such as mTOR/p70S6K and AKT/ERK. It is worth noting that relevant research on the combination of bitter melon vesicles with existing chemotherapy treatments and the incorporation of delivery via nanoparticles has also been investigated, exhibiting inhibitory activities in cell proliferation and augmenting oxidative stress. Key mechanisms identified in this review of in vitro studies are presented in Figure 1 and will be discussed in the following segments of the manuscript.

#### 3.1.1. Bitter Melon Compounds

Triterpenoids are a subclass of terpenoids, a large class of bioactive phytochemicals naturally occurring in a vast variety of foods [23]. Cucurbitane-type triterpenoids are a major class of bioactive compounds present in bitter melon. The terpenoids of *Momordica charantia* L. have been investigated in a variety of studies for their antitumor activity (Table 2). Namely, the triterpenoid 3β,7β,25-trihydroxycucurbita-5,23(E)-dien-19-al isolated from the plant has been studied against breast cancer cell lines (MCF-7 and MDA-MB-231), exhibiting suppressive activity against the proliferation of cancer cells within 72 h of treatment (IC_50_: 19 and 23 μM, respectively). This 2016 research also highlighted the compound’s potential to activate PPAR-γ isoform, while also participating in biological modulations, including the downregulation of Akt-NF-κB signaling, the upregulation of p38 MAPK and p53, increased ROS generation, the inhibition of histone deacetylase (HDAC) protein expression, and cytoprotective autophagy [24]. The antiproliferative potential of this compound has also been reported in evaluations of the human tongue SAS (squamous cell carcinoma) cell line, with limited potential at a concentration of 20 μM, and relatively higher at 40 μM, during treatment for 24 h [25]. The cytotoxic potential against breast cancer cells (MCF-7 cells, IC_50_ of 10 μM) as well as the PPAR-γ activating potential have also been reported for 5β,19-epoxy-19-methoxycucurbita-6,23-dien-3β,25-diol, another triterpenoid compound isolated from the plant [26]. Also, (19R,23E)-5β,19-epoxy-19,25-diethoxycucurbita-6,23-dien-3β-ol (named charantoside XIII), a new cucurbitane-type triterpene isolated from the fruit, exhibited significant cytotoxic activity against HeLa cells, with an IC_50_ value of 11.18 μM, while also exhibiting cytoprotective effects against H_2_O_2_-induced injury in MIN6 β-cells at a concentration of 10 μM [27]. In a similar setting, investigating potential herbal PAK1-inhibitors (PAK1 or RAC/CDC42-activated kinase 1, is a major oncogenic kinase), an early study of 2016 tested the effects of cucurbitacin I on the growth of cancer or normal hair cells, and melanogenesis in the cell culture of A549 lung cancer, and B16F10 melanoma, highlighting its potential to inhibit the growth of human lung cancer cells with an IC_50_ of 140 nM [28].

Similarly, a 2019 study examined the anti-hepatic fibrosis activity of cucurbitane triterpenoid glycosides against liver cancer cell lines HepG2 (hepatocellular carcinoma) and Hep3B. Namely, karaviloside III had antiproliferative activity with IC_50_ (μM) values of 4.12 ± 0.27 and 16.68 ± 2.07, 19(R)-5β,19-epoxycucurbita-6,23-diene-3β,19,25-triol had antiproliferative activity with IC_50_ values of 84.72 ± 6.76 and 32.55 ± 3.68, and karavilagenin B had antiproliferative activity with IC_50_ values of 24.76 ± 2.9 and 68.42 ± 4.97 after 48 h of incubation with the compounds [29]. Along these lines, cucurbitane-type triterpenoids of the plant have also been reported to have cytotoxicity potential against the MCF-7, HEp2 (laryngeal carcinoma), HepG2, and WiDr (colon adenocarcinoma) cancer cell lines. Compounds such as kaguaovin K (IC_50_ values at 9.45 ± 0.51, 9.66 ± 0.34, 8.69 ± 0.46, and 9.23 ± 0.04 μM), followed by kaguaovin I (IC_50_ of 20.94 ± 0.44 μM in WiDr) and kaguaovin H (IC_50_ of 16.71 ± 0.78 μM in HepG2) have had moderate cytotoxicity after 24 h of incubation, but kaguaovin K (10 μg/mL) has also demonstrated anti-inflammatory activity (against NO production in LPS-stimulated cells) of 92.81 ± 1.66% [30].

Another spirohydantoin-containing cucurbitane-type triterpenoid, kaguacidine A, was also evaluated for anti-inflammatory activity (against NO production in LPS-stimulated RAW 264.7 cells) and anti-proliferative activity against HEp2, MCF-7, HepG2, and WiDr cells. The results of this 2023 study reported moderate anti-inflammatory (IC_50_ of 18.5 ± 0.4 μg/mL) and anti-proliferative activities (IC_50_ of 31.0 ± 0.7, 33.8 ± 0.6, and 27.0 ± 0.7 μM in the aforementioned cell lines, respectively) [31]. Kuguacin J is another compound investigated in A2780 cells (chemotherapeutic drug-sensitive human ovarian carcinoma) and SKOV3 cells (ovarian cancer cell line) in an effort to evaluate its ability to promote cisplatin- and paclitaxel (PTX)-induced cancer cell death after 48 h of treatment. The results of this study demonstrated that Kuguacin J treatment (at a concentration of 30 μM) reduced the growth of A2780 cells, with an IC_50_ of 18.3 µM, but had little effect on promoting cisplatin’s and paclitaxel’s action. In the case of SKOV3 cells, the treatment (at 30 μM) reduced cell growth, with an IC_50_ of 43.33 ± 5.77, and was able to enhance the cytotoxicity of PTX but not increase cisplatin sensitivity [32]. Momordicine-I, another bioactive metabolite of *Momordica charantia*, has been investigated in the setting of head and neck cancer cells (JHU022, JHU029, Cal27), demonstrating inhibitory potential in cell viability in a dose-dependent manner, with an IC_50_ value of 10.4 μg/mL, while also inhibiting c-Met and its downstream signaling molecules c-Myc, survivin, and cyclin D1 through the inactivation of STAT3 [33].

Two key ribosome-inactivating proteins (RIPs) produced by *Momordica charantia*, namely, alpha-Momorcharin (α-MMC) and momordica anti-human immunodeficiency virus protein (MAP30), have been investigated for their inhibitory effects on cultured tumor cells. MAP30 has been investigated in a variety of settings, and key findings include its potential to inhibit the cell proliferation and clone formation of 5637 (bladder carcinoma cell line) and T24 (human bladder cell line) cells by promoting apoptosis and cell cycle arrest [34], while, in the case of T24 cells, reports also highlight that the treatment was able to concentration-dependently inhibit the proliferation and migration of cells with an IC_50_ of 320.48 μg/mL [35]. Additionally, MAP30’s inhibitory potential is linked to the suppression of the AKT (or PI3K-Akt) and EMT (epithelial–mesenchymal transition) signaling pathways [34], while MAP30 treatment was also reported to downregulate the expressions of NF-kB, JNK, and MMP2 and reduce their migratory ability [35]. In the setting of the HL-60 (promyelocytic cell line derived from human leukemia) and THP-1 (human monocytic leukemia cell line) cell lines, MAP30 treatment (48 h) was able to inhibit cell proliferation, with IC_50_ of 2.6 and 9.2 µM, respectively, through autophagy inhibition and apoptosis induction. Notably, MAP30-induced apoptosis was also related to PARP proteins’ and survivin’s decrease (also reported in the evaluation of momordicine-I, in HNC cells [33]), as well as to the activation of caspases, including the initiator caspase-8 and caspase-9, and their upregulation, as revealed in further testing. These results were also relevant in the setting of primary AML (acute myeloid leukemia) cells isolated from the bone marrow of newly diagnosed and untreated patients, further highlighting the caspase-dependent manner of MAP30-induced apoptosis [36]. The effect of MAP30 treatment on apoptosis-related proteins has also been documented in its evaluation in HCC-LM3 (hepatocellular carcinoma) cells. In this context, in addition to the inhibitory potential of the treatment in cell proliferation and survival, the results report the downregulation of PARP-1 and caspase-3 and the upregulation of activated caspase-9 within 24 h of treatment [37].

Several studies have examined the effect of α-MMC on the inhibition of various cancer cell lines, closely related to its cytotoxicity as well as anti-inflammatory potential. Namely, the inflammatory responses induced by recombinant α-MMC (purified from *Escherichia coli*) were evaluated in human THP-1 monocytic cells at a dose of 40 µg/mL. The 24 h treatment exerted an LPS (1 µg/mL) cytotoxic effect on THP-1 monocytic cells, while various cytokines and proinflammatory proteins, including IL-6, IL-1β, and TNF-α, were upregulated (with fold change > 2, as reported in the study). Also, the treatment was able to activate the IKK/NF-κB pathway (IκBα degradation at 60 min, followed by a gradual IkBα restoration at 120 min, in an overall evaluation up to 4 h, as reported in the study), as indicated by the increasing p65 protein levels in the nucleus, while increased levels of phosphorylated JNK also highlight the key role of the JNK pathway in α-MMC-induced inflammatory responses [38]. Working also with THP-1 cells, but on a higher dose of α-MMC, a study conducted the same year reported a significant effect of apoptosis induction in THP-1 cells. Namely, the doses of 20 μg/mL, 40 μg/mL, 80 μg/mL, 120 μg/mL, and 160 μg/mL were evaluated, showing that α-MMC at doses higher than 80 μg/mL were able to induce significant apoptosis within 24 h of treatment. Also, the α-MMC treatment had an impact on the expression levels of cytokines with synthesis promotion and synthesis exhibition effects reported by the researchers. Namely, during the early phase (2–8 h), it promoted the synthesis of IL-8, IL-9, MIP-1α, MIP-1β, and MCP-1, while in the later phase (8–24 h), it suppressed their expression. Additionally, α-MMC treatment led to sustained activation of IL-1ra and RANTES, while persistently inhibiting the expression of IL-1β and TNF-α [39]. Such effects of α-MMC-induced apoptosis and regulation of inhibitory cytokine expression are also supported by another study conducted in 2019 [40]. This study, which included evaluations involving animal models, will be further elaborated upon in the segment dedicated to animal studies.

Additionally, investigating A549 (adenocarcinoma human lung epithelial cells) and HepG2 cell lines, previous research documented that α-MMC treatment at concentrations of 0.5, 1, 2, 4, and 8 µM for 72 h resulted in increased cell growth inhibition in a dose-dependent manner, reaching approx. 70% at the higher dose [41]. Also, in the setting of breast cancer (MCF-7 cells), α-MMC was evaluated in different concentrations (ranging from 210.0 μg/mL to 3.3 μg/mL), underscoring inhibitory potential on cell growth in a dose-dependent manner, with an IC_50_ of 33.66 μg/mL, while also reporting a time dependency in inhibiting cell growth (as the test was carried out for 24, 48, and 72 h, showing increasing effects). In a similar manner, caspase-3 activities have been reported to increase after treatment (210 μg/mL for 48 h), therefore linking them to higher apoptotic action [42].

#### 3.1.2. Bitter Melon Extracts

A variety of extracts (crude juice or via solvent extraction) derived from this natural product have also been an item of investigation for their ability to modulate cellular mechanisms, including autophagy, apoptosis, and oxidative stress, across various cancer cell lines. In this segment, key outcomes of such studies investigating bitter melon extracts are presented and briefly summarized in Table 3. Namely, bitter melon extracts (directly extracted from bitter melon without seeds) were evaluated in MCF-7 cells for 12, 24, and 48 h at a concentration of 2% *v*/*v*, resulting in the induction of autophagy in the cells in a time-dependent manner. The goal of this study was to support that autophagic cell death of MCF-7 cells is possible due to treatment with the extract. As reported in the study, the treatment was able to accumulate protein p62 expression in a time-dependent manner in MCF-7 cells as compared to untreated cells, thus providing a potential link to apoptotic cell death [43]. In a similar setting, also using the direct extract of the natural product at a concentration of 2% *v*/*v* in MCF-7 cells, the treatment significantly reduced cell viability at 48 h of treatment (at approx. 95%, as extracted from the graphical data of the study) in cells cultured in high glucose (450 mg/dL) [44].

Working on a different breast cancer cell line, MDA-MB-436, and on A549 cells, a recent 2021 study also investigated bitter melon extracts; however, in the form of aqueous extracts initially prepared and then fractionated using ethyl acetate, n-hexane, and dichloromethane. The concentrations of the fractions used in the cell treatments were 100 and 125 μg/mL for a treatment period of 24 h. The results of this study highlighted that the dichloromethane fraction was the most potent at a concentration of 125 μg/mL, as it was able to significantly reduce the mitochondrial membrane potential at 43.08 ± 2.33, compared to the untreated cells, 131.7 ± 11.21. Notably, the study highlights that no biochemical and morphological apoptotic features were observed in any of the treatments, including failure to activate caspase-3 or cytochrome c release, although there has been an increase in ROS levels and an energetic decrease (reflecting ATP levels) [45]. Similar results of increased ROS levels and decreased mitochondrial membrane potential are reported in another 2021 study evaluating the effects of the water extract of the fruit on HepG2 cells. Namely, ROS levels were increased by 1.3-fold and 1.6-fold when treated at concentrations of 600 µg/mL and 1000 µg/mL, respectively, while mitochondrial membrane potential was decreased by 0.55-fold compared to the control when treated at a concentration of 600 µg/mL. Additionally, treatment duration was 24 h, and it was able to inhibit the proliferation of HepG2 cells, reduce cell viability in a dose-dependent manner (IC_50_ of 586.27 ± 0.07 µg/mL), and induce apoptosis. Notably, the miR-421 expression level was also downregulated by 13.74-fold at a treatment concentration of 600 µg/mL [46].

Two recent studies have been designed to investigate the fruit’s extract, focusing on two common varieties (the Chinese and the Indian bitter melon) in the setting of cancer. The first worked with A549 cells treated with hot and cold aqueous extracts (treatment concentration ranging from 1000 μg/mL to 100 μg/mL, for a treatment period of 24 h) from both varieties, reporting that the hot aqueous extracts (IC_50_ values of 32.5 ± 0.18 and 36.9 ± 0.08 for the Chinese and Indian varieties, respectively, compared to cisplatin 17.3 ± 0.01) were able to inhibit cell proliferation activity (possibly related to a 5-fold increase in ROS activity linked to mitochondria injury) and increase the caspase-3/7 activity by 1.6-fold, compared to untreated cells (or reach 61.64% and 62.68% increase, respectively) [47]. The second worked with PANC-1 cells (pancreatic adenocarcinoma) treated with lyophilized juice of the Chinese variety (treatment concentrations of 2% and 4% *v*/*v*) and aqueous-methanolic extracts of the Indian variety (treatment concentrations of 2%, 4%, and 6% *v*/*v*). The results of this study report that both varieties and extract preparations were able to inhibit PANC-1 cell proliferation and induce cell death in a dose-dependent manner. Namely, the juice treatment at 2% and 4% *v*/*v* for 72 h led to approx. 49% and 90% decreases in the total number of PANC-1 cells, respectively, whereas the extract preparations had limited effects on cell death [48]. It is worth noting that an earlier study investigating the effects of bitter melon juice on pancreatic cancer has also reported outcomes on the MiaPaCa2, PANC-1, and AsPC1 cell lines. Namely, treatments of 2% and 4% were able to decrease the total cell number in all cell lines after treatment for 72 h: MiaPaCa2 (by 83% and 92%, respectively), PANC-1 (by 69% and 97%, respectively), and AsPC1 (by 80% and 91%, respectively). The treatment was also reported to induce cell death in a dose-dependent and time-dependent manner in PANC-1 and AsPC1 cells (higher effects at 72 h), whereas in the case of MiaPaCa2 cells, cell death was also achieved at 24 h (PANC-1 and AsPC1 cells had lower percentage of dead cells compared to the untreated controls) [49].

In a similar setting, a number of ovarian cancer cell lines (A2780cp, A2780s, C13*, and OV2008) and human immortalized epithelial ovarian cells (HOSE17-1 and HOSE 96-9-18) were treated with different concentrations (0%, 0.25%, 0.5%, and 1%, *v*/*v*) of bitter melon extract (crude extract from the fruit—filtered, but no particular solvents were used). The treatment was able to inhibit cell growth in all cancer cell lines at a range of 20% to 87% compared with controls, but such an effect was not reported in the case of HOSE17-1 cells. As such, the researchers proposed that the extract treatment could mimic the action of a pharmacological activator of AMPK by inhibiting the growth of cancer cells, while limiting the cytotoxic effects on normal ovarian epithelial cells. In an effort to verify this hypothesis, further tests revealed that treatment with either 5% *v*/*v* or 10% *v*/*v* of the extract was able to increase cleavage of PARP and caspase-3 in C13* and OV2008 cells, thus highlighting the apoptotic potential, which was primarily relevant in the evaluations of specific compounds of bitter melon, as previously described [50].

#### 3.1.3. Bitter Melon Derivative Combination Techniques

The derivatives of bitter melon as well as crude extracts, extracellular vesicles (EVs), and fusion proteins, demonstrating promising cytotoxic and apoptotic effects across diverse cancer cell lines, have also been investigated in the setting of advanced approaches, such as encapsulation in liposomes and the green synthesis of nanoparticles, and co-treatment with known pharmaceuticals, etc., to enhance their therapeutic potential. Such findings of previously conducted studies are scrutinized in this segment and briefly summarized in Table 4.

In an effort to enhance the effects of momordica charantia lectin, a type II RIP of bitter melon, against hepatocellular carcinoma cells (HepG2 and PLC/PRF/5), an early 2016 study reported that at 24 h exposure, cell treatment at 2.5 μM and 5.0 μM was able to induce cell death, in both cell lines (by approx. 20–30% reduction in cell viability in HepG2 and 40–50% in PLC/PRF/5 cells) while also decreasing the colony formulation potential of cells in terms of number and size. Additionally, a concentration-dependent increase in apoptotic cells was reported upon treatment at either concentration (2.5 and 5.0 μM), with apoptotic cells recorded at 11.6%  ±  2.5 and 19.6%  ±  3.3, respectively. Notably, the co-treatment of cells with both *Momordica charantia* lectin and sorafenib (concentrations of 1.25 μM and 5 μM, respectively) was able to further decrease the cell viability (compared to the single use of *Momordica charantia* lectin or sorafenib at the same concentrations) and increase the sorafenib-induced apoptosis by 3.37 and 3.49 folds in HepG2 and PLC/PRF/5 cells, respectively [51]. Similar efforts explored the potential of bitter melon crude extract in enhancing the antitumor activity of natural killer (NK) cells in the setting of head and neck squamous cell carcinoma (Cal27 or JHU-29 cell lines). The results report that treatment with the extract at a concentration of 1% *v*/*v*, over 24 h, was able to improve NK-cell-mediated tumor killing, in which case the tumor cell/effector cell ratio has been relevant to the intensity of the effect (ratios tested were 1:25, 1:10, and 1:1). Additionally, higher granzyme B expression was observed in the extract-treated NK cells as compared to untreated cells, which was also reported for NK cells co-cultured with either Cal27 or JHU-29 cells [52].

Plant-derived extracellular vesicles (PDEVs) have gained attention for their potential in medicine, particularly as natural, biocompatible carriers for drug delivery. As such, the EVs of bitter melon were evaluated in their potential to improve the effects of 5-Fluorouracil against oral squamous carcinoma (Cal27 and WSU-HN6) cells. In this setting, the results demonstrated a dose-dependent (EV concentrations ranged from 4 to 20 μg/mL) reduction in cell viability in either cell line, reaching up to 80% in higher concentrations, thus highlighting the EVs’ ability to modulate biological processes. In the evaluations of the synergistic effect of EVs and 5-Fluorouracil, results also report a significant reduction in cell viability, which was more pronounced in Cal27 cells (reaching up to approx. 80%) in addition to higher apoptotic ratios as compared to the single 5-Fluorouracil treatment. It is also worth noting that the single 5-Fluorouracil treatment upregulated the pro-IL-1β expression as compared to the control in both cell lines; however, the synergetic effect with EVs was able to significantly downregulate the pro-IL-1β expression. These results were also relevant and exhibited similar trends in the case of NLRP3 (NOD-like receptor protein 3) expression [53]. In a similar setting, also investigating the EVs of bitter melon, but working with 4T1 (murine breast cancer) and MCF-7 cells, a recent 2023 study demonstrated that treatment at a concentration of 20 μg/mL effectively inhibited cell proliferation in a time-dependent manner, with initial results being observed at 12 h post-treatment. Furthermore, the treatment suppressed 4T1 cell migration (by approx. 15% at 24 h) and colony formations, compared to the untreated cells, as well as induced apoptosis of approx. 21% at 20 μg/mL in both cell lines treated for 24 h. Notably, a higher concentration of 40 μg/mL for a longer treatment period (48 h) could result in about 55% apoptosis, with the effect being more pronounced in 4T1 cells compared to MCF-7 cells [54].

As previously described, key RIPs of bitter melon, such as α-MMC and MAP30, have very effective cytotoxicity rates against various cancer cell lines. However, insufficient cellular internalization in cancer cells is a limiting factor for their potential. In this setting, the development and utilization of cell-penetrating peptides, namely a heparin-binding domain derived from heparin-binding epidermal growth factor, was utilized in an effort to investigate their potential to enhance the cytotoxicity of α-MMC and MAP30 in two different studies. In the first one, an α-MMC-HBP fusion protein was tested on HeLa (human cervical cancer), MGC80-3 (human gastric cancer), MCF-7, and 95D (human lung cancer) cells. The results demonstrated significantly enhanced cytotoxic effects in a dose-dependent manner (the concentrations evaluated were 0.25, 0.5, 1, 3, and 5 μM). Namely, at 48 h of treatment, IC_50_ values of α-MMC-HBP fusion protein were 0.608 μM, 0.385 μM, 0.221 μM, and 1.392 μM, for HeLa, 95D, MCF-7, and MGC80-3 cells, respectively, which were rendered much lower than those observed for the single α-MMC treatment (7.191 μM, 71.640 μM, 3.206 μM, and 4.062 μM, respectively). This improved cytotoxic ability was correlated to the higher level of cell apoptosis induced by α-MMC-HBP, which was relevant to the activation of caspase-8, caspase-9, and caspase-3, more keenly than α-MMC alone and upregulation of PARP, as reported by the researchers [55]. In a similar way, the MAP30-HBP fusion protein (treatment concentrations of 0.25, 0.5, 1, 3, and 5 μM) was investigated against HeLa, 95D, B16, and MCF-7 cells (treated for 24 h), and MGC803 and HepG2 cells (treated for 48 h). The results demonstrated a significantly enhanced cytotoxic effect as compared to the single MAP30 treatment in all of the tested cell lines. Namely, the IC_50_ values were 0.34 ± 0.024 μM (with a 28.18-fold difference) on HeLa cells, 1.15 ± 0.107 μM (with a 145.13-fold difference) for 95D cells, 0.18 ± 0.041 μM (with a 157.83-fold difference) for B16 cells, 0.86 ± 0.101 μM (with a 7.73-fold difference) for MCF-7 cells, 0.02 ± 0.005 μM (with a 200.00-fold difference) for MGC803 cells, and 0.09 ± 0.012 μM (with a 102.00-fold difference) for HepG2 cells, respectively.

Further investigations carried out for HeLa cells demonstrated that the treatment with MAP30-HBP at concentrations of 0.5 μM, 1 μM, and 2 μM was able to increase apoptosis potential by approx. 30%, as compared to single MAP30 treatments of the same concentrations, leading to the activation of caspase-3, caspase-8, and poly-ADP ribose polymerase (PARP). Additionally, MAP30-HBP treatment decreased the expression of Bcl-2 but had little effect on the expression of Bax, thus increasing their ratio, indicating that apoptosis in HeLa cells was linked to the mitochondrial- and death receptor-mediated signaling pathways [56]. Similar results were obtained in a recent 2018 study, also in the setting of HeLa cells, where the MAP30 RIP was fused with S3 (MAP30-S3), an epidermal growth factor receptor (EGFR)-targeting peptide, in an effort to determine the added value to its effect when used with cyclosporin A. The result reported that treatment with 2 μmol/L cyclosporin A significantly increased the cytotoxicity of MAP30-S3 on HeLa cells with an IC_50_ of 40.3 nmol/L (approx. 125-fold difference compared to single MAP30-S3 treatment) and was able to increase its initial apoptotic activity by 77.55% [57]. In a more recent 2021 study, the EGFR-targeting peptides were again relevant for combination with MAP30 in the setting of HepG2, MDA-MB-231, HUVEC, and MCF-7 cells. Namely, the study aimed to evaluate the added value to the MAP30’s activities when modified with an RGD motif and an EGFRi motif. The recombinant protein demonstrated high cytotoxicity in all evaluated cell lines, with IC_50_ values of 54.64 μg/mL, 70.13 μg/mL, 146 μg/mL, and 466.4 μg/mL, respectively. Additionally, the treatment had a similar effect as previously described on the Bcl-2 to Bax ratio, with Bax being upregulated, particularly in HepG2 cells, thus indicating the effective induction of apoptosis [58].

An interesting area of research over the past years has also been related to advanced nanomaterial approaches and green synthesis methods in cancer treatment, showcasing their potential for improving the existing characteristics of various nutraceuticals. As such, MAP30 has been encapsulated in liposomes and evaluated against T24 cells in an effort to validate a potential improvement in its cytotoxicity. The outcomes of the MAP30 liposomes indeed highlight an increased inhibitory effect on cell proliferation, with an IC_50_ value of 51.06 μg/mL, as compared to that of MAP30 (IC_50_: 320.48 μg/mL) [35]. In a similar setting, an eco-friendly green approach has been previously introduced to synthesize stable, cytotoxic colloidal silver nanoparticles (NPs) loaded with the crude fruit extract of bitter melon (25 mL of extract was added to 100 mL of 1 mM AgNO_3_ solution). The NPs were evaluated against A549, and the results demonstrated an increased cytotoxic potential of the loaded NPs with an IC_50_ value of 51.93 µg/mL compared to the crude extract’s (IC_50_ value of 102 mg/mL) [59].

### 3.2. Animal Models

**Figure 2:**
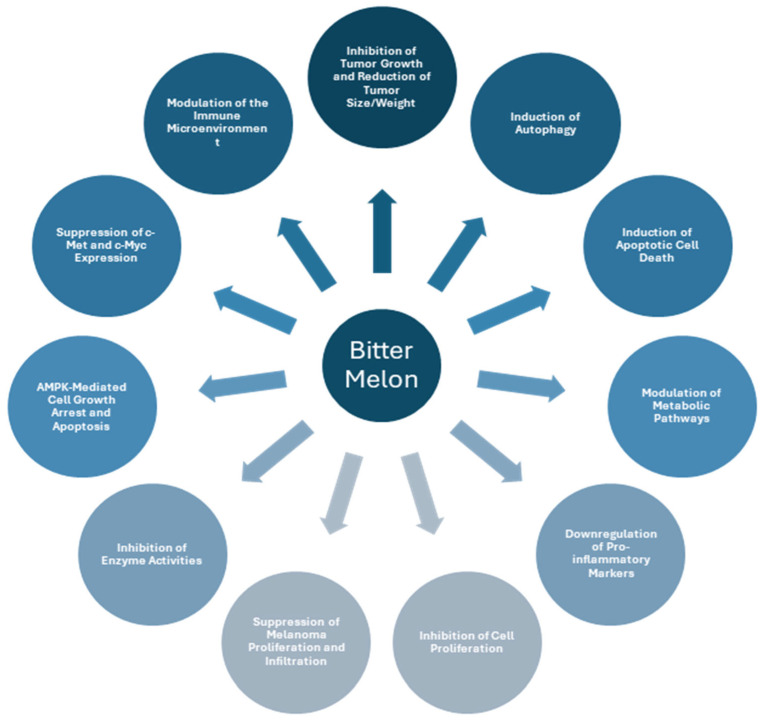
Key mechanisms identified in *animal* studies.

Several remarkable findings of the in vitro studies have been further validated in animal studies settings, mainly in mice and rat models. This section explores animal studies assessing bitter melon extracts, derivatives, and synergistic effects when combined with other natural or pharmaceutical compounds. Key mechanisms identified in the animal models presented in Figure 2 are discussed in this segment. Notably, characteristics such as the inhibition of cell proliferation established in several in vitro studies are validated by showing the inhibition of tumor growth and a reduction in tumor size or weight in various animal models. Similar outcomes, such as the modulation of inflammatory markers and the inhibition of enzymes, primarily identified in vitro, are also present in corresponding animal studies. By analyzing these investigations, we aim to summarize and holistically approach their biological activity and implications for future applications. A brief summary of the outcomes is presented in Table 5.

### Bitter Melon Derivatives and Extracts

Some of the previously described interventions, such as bitter melon crude extract and juice, as well as key derivatives such as MAP30, α-MMC, and momordicine-I, have been further investigated in animal models against various cancers. In fact, a recent 2019 study investigated the impact of bitter melon juice exposure on animals (Athymic nu/nu mice) injected with PANC-1 cells. This investigation consisted of three animal groups. The first group was initiated on juice treatment (200 mg/kg) for 5 days/week, 24 h post-injection with PANC-1; the second group (also injected) was allowed 2.5–3 weeks for the tumors to grow; and after that, the animals were either randomized into the control group or the third group, where they received juice treatment (200 mg/kg) for approx. 7 weeks. The tests were conducted at baseline, on day 7 after randomization, and at the end of the study. The outcomes of this study demonstrated that the apparent diffusion coefficient values (measured via MRI scan) taken on day 7 were increased in all study groups (control group (1.05 ± 0.33, ×10^3^ mm^2^/s), group one (1.56 ± 0.03, ×10^3^ mm^2^/s), and group three (1.48 ± 0.04, ×10^3^ mm^2^/s)). However, during the end-of-study measurements, the values for all groups dropped (control group (0.92 ± 0.11, ×10^3^ mm^2^/s), group one (1.15 ± 0.08, ×10^3^ mm^2^/s) and group three (1.12 ± 0.25, ×10^3^ mm^2^/s)), with the intervention values remaining higher than those of the control, thus suggesting response to treatment and slower tumor growth. It is worth noting that throughout the course of the study, the body weight of the animals was monitored in order to verify any apparent toxicity of the treatment. As such, the study reports that no significant changes were observed. Additionally, detailed evaluations of tumor growth trends in all groups highlight that in both treatment groups, the tumor volume remained lower than the control’s and within the range of their initial size (established before the randomization) [60].

An earlier study, also investigating the effects of bitter melon extract (crude at concentrations of 0.25% and 10%) on the inhibition of ovarian cancer cell growth, employed ovarian cancer cells (ES-2) implanted into BALB/cAnN-nu female nude mice. Intraperitoneal administration of the extract was initiated 2 days after cell injection, and cisplatin treatment was introduced every other day for a total of 6 injections (without stopping the extract treatment). The control group of this study received PBS injections instead of cisplatin, and tumor size was monitored after each injection in both groups. The results demonstrated reductions in tumor weights at the levels of 40% to 70% in both treated groups by day 12, as compared to the control group [50]. Also working with bitter melon extract in the setting of breast cancer, a 2017 study reported on the extract’s inhibitory potential, as the treatment (30% *v*/*v*, 600 mg/mouse/day) was able to decrease breast cancer growth in mouse models (Nude, Balb/c, and C57BL/6 mice—syngeneic and xenograft setting). The treatment was initiated a day after tumor cell implantation and continued for approx. 4 weeks (study end). The control group of the study received the same volumes of water instead of the extract. Notably, the researchers documented that the tumor growth rate was lower in the treatment group and resulted, at the end of the study, in smaller-weighted tumors (approx. 50% lower than the control group, as extracted from the study’s graphs). In addition, these effects were closely related to further findings on increased p62 accumulation in tumor lysates (almost 2-fold increase), the induction of autophagy (as previously related to PARP cleavage in cancer cells), and the induction of apoptotic cell death in the treatment group [43].

Evaluating the effects of bitter melon extract on the progression of head and neck squamous cell carcinoma (oral), two recent studies have worked on either 4-nitroquinoline 1-oxide carcinogen-induced cancer in C57BL/6 mice or subcutaneous injection of cells into the flanks of syngeneic mice (C3H/HeNTac). In the first setting, treatment with the extract was 30% *v*/*v*, 600 mg/mouse, or water for the control group for a period of 16 weeks. The findings demonstrated a significant reduction in the incidence of induced oral cancer in the treatment group as opposed to the control group, which showed neoplastic changes ranging from moderate dysplasia to invasive squamous cell carcinoma. Also, the treatment group was able to continue to gain body weight until the end of the study. Notably, pro-inflammatory molecules such as the s100a9, IL23a, IL1b and PD1 genes, displayed downregulation of 3.02-fold, 7.57-fold, 5.99-fold, and 1.7-fold, respectively [61]. In the second setting, the treatment was 100 μL of the extract for 5 days/week, for a period of 4 weeks. Similarly to the previous study, the treatment significantly inhibited tumor growth as compared to the control group (which received water), while the difference between the groups was significant after day 15 of the experiment. Also, cell proliferation was inhibited, as evident by the low expression, at almost 50% compared to the control group of PCNA (proliferating cell nuclear antigen) and Myc-oncogene (a key regulator of tumor microenvironment) in the treatment group [62].

A recent 2024 study aimed to investigate the inhibitory potential of a bitter melon extract in the metastasis of melanoma cells in the lungs of C57BL/6j mice. The animals were injected with melanoma cells, and the extract was orally administered daily (50 mg/kg, three times per day) for 2 weeks. The study groups included untreated healthy mice, mice with melanoma but without treatment (control), and the treated mice (intervention). As reported by the study, the intervention was able to ameliorate melanoma proliferation and infiltration into the lungs, with DOPA-positive cells being more than 2-fold fewer in the treatment group. Also, the reduction was closely related to the suppression of PAX3 (paired box gene 3) expression, as compared to the control group (mice with melanoma but not treated with the extract) and, thus, the regulation of PTEN/PI3K/Akt signaling [63]. In a similar setting, working with albino western rats, researchers also aimed to investigate a bitter melon extract’s (methanol) potential in inhibiting cell proliferation, angiogenesis, and metastasis in hepatocellular carcinoma. The study included a diethyl nitrosamine injection (200 mg/kg body weight once); subcutaneous treatment with carbon tetrachloride (3 mL/kg/week) after 2 weeks (continued for 10 weeks); and oral administration of the extract (40 mg/kg) in groups before, at the time of, and after the treatment with diethyl nitrosamine. The entire study was concluded within 16 weeks, and the extract treatment was continued until that time (for the respective treatment groups). The outcomes of this study highlight that the treatment was able to decrease COX-2 (cyclooxygenase-2) activity as expressed by the levels of prostaglandins, with lower concentrations in the treatments before and after diethyl nitrosamine, rather than the group of simultaneous treatment; VEGF (vascular endothelial growth factor) at a similar manner to that with prostaglandins but with lower intensity of differences between the treatment groups; HDAC, with 45%, 31%, and 42% reductions before, during, and after diethyl nitrosamine treatment, respectively; and MMP-2 and MMP-9 (matrix metalloproteinases), with MMP-9 being more affected than MMP-2 and reaching reduction levels of 54%, 40%, and 50% before, during, and after diethyl nitrosamine treatment, respectively, as compare to the untreated group. Additionally, the expression levels of caspase-3 and caspase-8 were elevated in all treatment groups, indicating the activation of apoptosis, with during treatment having the lowest effect among them [64].

As previously described, MAP30 administered with cisplatin produced a synergistic effect on cisplatin-induced cell cytotoxicity in ovarian cancer cells. Similar results of inhibitory potential in the proliferation of cells and tumor growth are reported in the testing of BALB/cAnN-nu female nude mice, when cisplatin and MAP30 (250 μg/kg, once every 2 days) were co-injected. Notably, AMPK-mediated cell growth arrest and apoptosis, which are relevant when bitter melon extracts were used in either in vitro or animal settings, was also reported in this study. Namely, an approx. 1.5-fold reduction was observed in the number of tumor nodules of the treatment group compared to the control group, while the ability of the animals to gain body weight was not affected. It is worth mentioning that this study took into account the key challenge related to the toxicity of MAP30 in higher doses and thus also evaluated a dose of 500 μg/kg in the animal model. In this case, the treatment group exhibited an approximate 10% weight loss in addition to the observation of minor liver damage [65]. In a different study, also working with MAP30, mice were transfected with bladder cancer cells for 48 h, in an effort to evaluate the inhibitory potential of the intervention on tumor growth, cell proliferation, the occurrence of metastasis, and its impact on the animals’ liver and kidneys. As the outcomes demonstrated, the treatment was able to inhibit tumor formation and reduce the tumor volume (estimated at a maximum of 25% based on the graphical presentations of the study), as compared to the control group. This observation was more pronounced after day 28 of treatment and until the end of the study (day 32). Histological examination showed that MAP30 induced mild histological changes in the liver and kidneys of mice; however, evaluations were reported only until day 7 of the study [35].

A recent 2024 study investigated the effect of momordicine-I treatment (30 mg/kg/mouse) in a mouse (C57BL/6 mice) head and neck cancer tumorigenicity model (MOC2 cell injection). The outcomes of this study demonstrated that treatment was able to significantly inhibit tumor growth, at an estimated 50% at the end of the study (21 days), based on the graphical presentations of the study, without significantly altering the animals’ ability to gain body weight [66]. In a similar setting, a study from the same year also employed the same treatment (30 mg/kg/mouse, in C57BL/6 mice model, with MOC2 cells injection) and also established that the treatment was able to reduce tumor growth, with slight differences during the mid-study-period (14 days) in between studies, but a relatively similar reduction rate compared to the control group at the end of the study (20 days). Additionally, a smaller dose of 20 mg/kg was administered either via injection or orally to C57Bl/6 male mice, revealing that the treatment was not toxic for the animals and had a favorable pharmacokinetics profile, particularly in the case of injected treatment. In the same setting, both 20 mg/kg and 30 mg/kg of momordicine-I treatment were further administered for five days to two different treatment groups, compared to an untreated group and a group receiving bitter melon extract (30% in the animals’ drinking water). The outcomes demonstrated no significant weight loss on the sixth day and no toxic effects of the treatment. In the evaluation of therapeutic potential in head and neck cancer, the 30 mg/kg of momordicine-I treatment (up to 30 days) was compared to the bitter melon extract and the control group. The results reported reduced tumor growth in the momordicine-I treatment, which was lower than those in the extract treatment as well as the control, while a swift weight loss was documented from day 14 to day 20, but the animals were able to gradually regain weight again until the end of the study. Notably, the research also reports on a significant reduction in the expression levels of c-Met (or hepatocyte growth factor receptor (HGFR)) and its downstream molecule c-Myc (a proto-oncogene), relevant in both treatment groups (extract or momordicine-I) as compared to the untreated mice [33]. A recent 2024 study also investigated the therapeutic effects of momordicine-I, demonstrating the interplay of tumor-associated macrophages and momordicine-I in head and neck cancer progression. As reported by the research, the intervention (30 mg/kg/mouse) administered daily via injection to C57BL/6 mice for 20 days, was able to disrupt the advance of tumor-associated macrophages and diminish B cell populations, thereby potentially re-establishing effective immune surveillance against cancer cells [67].

## 4. Discussion

The increasing interest in naturally occurring compounds from food and their potential to be utilized for cancer treatment reflects the ongoing pursuit of therapies that can potentially complement or even enhance conventional approaches like chemotherapy and radiotherapy [68]. As the previous literature on various natural products has demonstrated, these plant-derived compounds can offer unique multi-targeted mechanisms, modulating processes within the tumor microenvironment such as inflammation, angiogenesis, apoptosis, and cell proliferation. Such characteristics may elevate them into promising agents with a capacity to enhance therapeutic efficacy while minimizing adverse effects, which is a key element in the research of management options in oncology [69,70].

Among these, bitter melon stands out due to its diverse bioactive composition, including MAP30, α-MMC, and momordicine-I, which have consistently demonstrated anti-inflammatory, pro-apoptotic, and anti-angiogenic properties across in vitro and animal studies. Its impact is particularly evident in suppressing tumor growth, inhibiting metastasis, and inducing cancer cell death. The versatility of bitter melon as an intervention is further underscored by its application across a broad spectrum of cancers. It is worth noting that the mechanistic insights provided by these studies reveal bitter melon’s ability to regulate apoptosis via caspase activation, enhance autophagy, and modulate markers of inflammatory and angiogenic pathways. These biological effects are complemented by findings on toxicity management, where derivatives of the plant achieve a balance between efficacy and safety, an essential factor for clinical application. As previously mentioned, bitter melon extracts constitute a conglomeration of bioactive compounds with anticancer activity. Several of them belong to the cucurbitane-type triterpenoid phytochemical class. In this respect, triterpenoid analogues are included in various natural products, and they have been shown to exert significant anticancer properties both in vitro and in vivo [71,72,73]. These phytochemical compounds could be used as lead compounds in drug design settings to synthesize drug-like derivatives with higher efficiency and target selectivity, as well as less toxicity and enhanced oral bioavailability [74]. Machine learning techniques, artificial intelligence methods, quantum computing, and late-stage functionalization present novel challenges in generative chemistry and drug discovery processes, which could be applied in the case of bitter melon phytochemicals like triterpenoid analogues [75,76,77,78]. Moreover, the identification of several novel bioactive lead compounds, including those in bitter melon, has recently been obtained using in silico approaches such as molecular docking and molecular dynamic simulations, which could be tested for their anticancer activity [79,80].

Although several technical barriers, such as the extraction of key derivatives from natural products or assays against molecular targets, often hinder the broader evaluation of natural compounds derived from food, research from the past decades documents a steady interest surrounding nutraceuticals [81,82,83,84,85]. It is essential to also highlight the concerns linked to these naturally derived food substances in the setting of regulatory frameworks that govern them [86,87]. Unlike conventional drugs, nutraceuticals are often marketed as dietary supplements, raising concerns regarding their clinical evaluations to assess efficacy, safety, or potential adverse effects and ultimately their long-term impact on health, particularly when used in high concentrations or outside conventional therapeutic contexts. While compounds like bitter melon exhibit promising bioactive properties, their safety profiles, including interactions with conventional cancer therapies, remain insufficiently studied.

In this setting, concerns regarding the indiscriminate use of *M. charantia* dry extracts should not be overlooked. Previous studies utilizing *Allium cepa* and Wistar rat test models have documented cytogenetic toxicity attributed to the extract, with chromosomal alterations observed in the *Allium cepa* assay—a direct method for assessing exposure to mutagens or carcinogens [88]. Additionally, maternal toxicity during the organogenesis period was noted, as evidenced by reduced water and feed intake and mean body mass gain. Despite these findings, reproductive outcomes and developmental toxicity assessments indicated minimal skeletal malformations in offspring. These observations underscore the need for cautious use and thorough evaluation of *M. charantia* dry extracts, particularly in clinical settings.

In closing, it is clear that based on the majority of pre-clinical evaluations, bitter melon emerges as a natural product with derivatives with favorable characteristics in the realm of cancer management. Its consistent anticancer properties, although not stemming from the vast numbers of studies as compared to other natural products, bridge the gap between experimental discoveries and clinical possibilities. As the journey from laboratory evaluations to clinical interventions unfolds, bitter melon is surely a plant worth remaining relevant in future research.

## Figures and Tables

**Table 1:** Inclusion criteria.

Criterion	Inclusion
Study type	In vitro (cell cultures) Animals (mice, rats)
Language	English
Publication date	January 2015–December 2024
Intervention	*Momordica charantia* extracts (aqueous, organic solvents) *Momordica charantia* isolated compounds
Outcomes	Antitumor activity (cytotoxicity/cell viability) Antitumor activity (modulation of tumor growth in animals)

**Table 2:** Summary of outcomes from interventions on bitter melon derivatives.

Compound Investigated	Dose or IC_50_	Cell Lines Tested	Key Outcomes
**3β,7β,25-trihydroxycucurbita-5,23(E)-dien-19-al**	19 μM (MCF-7), 23 μM (MDA-MB-231)	MCF-7, MDA-MB-231, SAS	Suppressed proliferation, PPARγ activation, Akt-NF-κB downregulation, apoptosis induction
**5β,19-epoxy-19-methoxycucurbita-6,23-dien-3β,25-diol**	10 μM	MCF-7	Cytotoxic, PPARγ activation
**Charantoside XIII**	11.18 μM	HeLa, MIN6 β-cells	Cytotoxic to HeLa, cytoprotective in MIN6 β-cells
**Cucurbitacin I**	140 nM	A549, Hair follicle dermal papilla cells, B16F10	Growth inhibition in lung cancer cells
**Karaviloside III**	4.12 μM (HepG2), 16.68 μM (Hep3B)	HepG2, Hep3B	Antiproliferative activity
**Kaguaovin K**	9.45–9.66 μM	MCF-7, HEp2, HepG2, WiDr	Moderate cytotoxicity, anti-inflammatory potential
**Kaguacidine A**	31–33.8 μM	HEp2, MCF-7, HepG2, WiDr	Anti-inflammatory, antiproliferative
**Kuguacin J**	18.3 μM (A2780), 43.33 μM (SKOV3)	A2780, SKOV3	Reduced ovarian cancer cell growth, enhanced paclitaxel cytotoxicity
**Momordicine-I**	10.4 μg/ml	JHU022, JHU029, Cal27	Inhibited viability, suppressed STAT3 signaling
**MAP30**	320.48 μg/mL (T24), 2.6 μM (HL-60), 9.2 μM (THP-1)	5637, T24, HL-60, THP-1, AML cells	Apoptosis induction, suppression of AKT/EMT pathways
**α-MMC**	20–160 μg/mL (THP-1), 0.5–8 μM (A549, HepG2), 33.66 μg/mL (MCF-7)	THP-1, A549, HepG2, MCF-7	Apoptosis induction, cytokine regulation, and caspase-3 activation

Bitter melon compounds investigated.

**Table 3:** Summary of interventions and outcomes of bitter melon extracts.

Extract Type	Dose or IC_50_	Cell Lines Tested	Key Outcomes
**Crude bitter melon extract (filtered)**	2% *v*/*v*	MCF-7	Induced autophagy, accumulated p62 protein, potential apoptotic link
	2% *v*/*v*	MCF-7 high glucose culture	Reduced cell viability (~95%) after 48 h
	0.25–1% *v*/*v*	A2780cp, A2780s, C13, OV2008, HOSE17-1, HOSE96-9-18	Inhibited cancer cell growth (20–87%), limited cytotoxicity in normal ovarian cells, increased PARP/caspase-3 cleavage
**Bitter melon juice**	2–4% *v*/*v*	MiaPaCa2, PANC-1, AsPC1	Decreased total cell number, dose- and time-dependent cell death
**Lyophilized juice (Chinese variety)**	2–4% *v*/*v*	PANC-1	Dose-dependent cell death (up to 90% reduction)
**Aqueous extract fractions (ethyl acetate, n-hexane, dichloromethane)**	100–125 μg/mL	MDA-MB-436, A549	Dichloromethane fraction reduced mitochondrial potential, increased ROS levels, no apoptotic features observed
**Aqueous extract**	600–1000 μg/mL (IC50: 586.27 μg/mL)	HepG2	Increased ROS, decreased mitochondrial membrane potential, apoptosis induction, downregulated miR-421
**Hot and cold aqueous extracts (Chinese and Indian varieties)**	100–1000 μg/mL (IC50: 32.5 μg/mL, 36.9 μg/mL)	A549	Inhibited cell proliferation, increased ROS (5-fold), increased caspase-3/7 activity
**Aqueous-methanolic extracts (Indian variety)**	2–6% *v*/*v*	PANC-1	Limited cell death effects

Bitter melon compounds investigated.

**Table 4:** Summary of outcomes for combination treatments.

Investigated Approach	Dose or IC_50_	Cell Lines Tested	Key Outcomes
**Momordica charantia lectin** + Sorafenib	2.5 μM, 5.0 μM	HepG2, PLC/PRF/5	Induced apoptosis, reduced colony formation, enhanced sorafenib-induced cytotoxicity
**Bitter melon crude extract** + NK cells	1% *v*/*v*	Cal27, JHU-29	Improved NK-cell-mediated tumor killing, increased granzyme B expression
**Bitter melon extracellular vesicles** + 5-Fluorouracil	4–20 μg/mL	Cal27, WSU-HN6	Enhanced 5-Fluorouracil cytotoxicity, reduced cell viability, modulated inflammatory markers
**Bitter melon extracellular vesicles**	20–40 μg/mL	4T1, MCF-7	Inhibited cell proliferation, suppressed migration, induced apoptosis
**α-MMC-HBP fusion protein**	0.25–5 μM	HeLa, MGC80-3, MCF-7, 95D	Enhanced cytotoxicity, increased apoptosis via caspase activation
**MAP30-HBP fusion protein**	0.25–5 μM	HeLa, 95D, B16, MCF-7, MGC803, HepG2	Strong cytotoxic effects, apoptosis activation via mitochondrial and receptor-mediated pathways
**MAP30-S3 fusion protein ** **+ Cyclosporin A**	2 μmol/L	HeLa	Increased cytotoxicity, enhanced apoptosis (77.55% increase)
**EGFR-targeting peptides combined with MAP30**	54.64–466.4 μg/mL	HepG2, MDA-MB-231, HUVEC, MCF-7	Induced apoptosis, upregulated Bax expression
**MAP30 liposomes**	IC50: 51.06 μg/mL	T24	Improved cytotoxic potential compared to MAP30 alone
**Colloidal silver NPs loaded with bitter melon extract**	IC50: 51.93 µg/ml	A549	Enhanced cytotoxicity compared to crude extract

Bitter melon compounds investigated.

**Table 5:** Summary of animal models.

Intervention Type	Mice Type and Cancer Setting	Dose and Intervention Duration	Key Outcomes
Bitter melon **juice**	Athymic nude mice, PANC-1 cells	200 mg/kg, 5 days/week, Group 1: 24 h post-injection for ~5 weeks, Group 3: ~7 weeks	Increased ADC values, indicating treatment response; slower tumor growth; no significant toxicity observed
Bitter melon **extract**	C57BL/6j mice, melanoma (lung metastasis)	50 mg/kg, 3 times/day for 2 weeks	Reduced melanoma proliferation and lung infiltration, suppressed PAX3 expression, regulated PTEN/PI3K/Akt signaling
	Nude, Balb/c, and C57BL/6 mice, breast cancer (syngeneic and xenograft)	30% *v*/*v*, 600 mg/mouse/day, ~4 weeks	Reduced tumor weight, induced autophagy and apoptotic cell death, increased p62 accumulation
	C57BL/6 mice, oral cancer induced by 4-nitroquinoline 1-oxide	30% *v*/*v*, 600 mg/mouse, ~16 weeks	Reduced oral cancer incidence, downregulation of pro-inflammatory markers
	C3H/HeNTac mice, oral cancer (subcutaneous tumor model)	100 μL, 5 days/week, ~4 weeks	Reduced tumor growth, PCNA and Myc-oncogene expression
Bitter melon **extract** (***methanol***)	Albino western rats, hepatocellular carcinoma	40 mg/kg orally, different timings (before/simultaneous/after treatment), 16 weeks	Decreased COX-2, VEGF, MMP-2/9 activity, activated caspase-3/8 (apoptosis)
Bitter melon **extract** + ***Cisplatin***	BALB/cAnN-nu female nude mice, ovarian cancer cells (ES-2)	Crude extract (0.25–10%) + Cisplatin every other day, 6 doses over 12 days	Reduced tumor growth
**MAP30** + ***Cisplatin***	BALB/cAnN-nu mice, ovarian cancer	250 μg/kg every 2 days, evaluated at higher dose (500 μg/kg)	Reduced tumor growth, synergistic effect with cisplatin, minor liver damage at higher dose
**MAP30**	Mice, bladder cancer	Administered after 48 h injection, ~32 days	Reduced tumor growth, mild histological changes in liver and kidneys
**Momordicine-I**	C57BL/6 mice, head and neck cancer (MOC2 cells)	20–30 mg/kg daily, 21–30 days	Reduced tumor growth, no significant weight loss, reduced expression of c-Met and c-Myc

Bitter melon compounds investigated.
